# Age patterns in overweight and wasting prevalence of under 5-year-old children from low- and middle-income countries

**DOI:** 10.1038/s41366-021-00911-5

**Published:** 2021-07-22

**Authors:** Luiza I. C. Ricardo, Giovanna Gatica-Domínguez, Inácio Crochemore-Silva, Paulo A. R. Neves, Juliana dos Santos Vaz, Aluisio J. D. Barros, Cesar Gomes Victora

**Affiliations:** 1 International Center for Equity in Health, Federal University of Pelotas, Pelotas, Brazil; 2 Postgraduate Program in Physical Education, Federal University of Pelotas, Pelotas, Brazil

**Keywords:** Risk factors, Nutrition

## Abstract

**Objectives:**

To describe how overweight and wasting prevalence varies with age among children under 5 years in low- and middle-income countries (LMICs).

**Methods:**

We used data from nationally representative Demographic and Health Surveys and Multiple Indicator Cluster Surveys. Overweight and wasting prevalence were defined as the proportions of children presenting mean weight for length/height (WHZ) more than 2 standard deviations above or below 2 standard deviations from the median value of the 2006 WHO standards, respectively. Descriptive analyses include national estimates of child overweight and wasting prevalence, mean, and standard deviations of WHZ stratified by age in years. National results were pooled using the population of children aged under 5 years in each country as weight. Fractional polynomials were used to compare mean WHZ with both overweight and wasting prevalence.

**Results:**

Ninety national surveys from LMICs carried out between 2010 and 2019 were included. The overall prevalence of overweight declined with age from 6.3% for infants (aged 0–11 months) to 3.0% in 4 years olds (*p* = 0.03). In all age groups, lower prevalence was observed in low-income compared to upper-middle-income countries. Wasting was also more frequent among infants, with a slight decrease between the first and second year of life, and little variation thereafter. Lower-middle-income countries showed the highest wasting prevalence in all age groups. On the other hand, mean WHZ was stable over the first 5 years of life, but the median standard deviation for WHZ decreased from 1.39 in infants to 1.09 in 4-year-old children (*p* < 0.001). For any given value of WHZ, both overweight and wasting prevalence were higher in infants than in older children.

**Conclusion:**

The higher values of WHZ standard deviations in infants suggest that declining prevalence in overweight and wasting by age may be possibly due to measurement error or rapid crossing of growth channels by infants.

## Background

In the recent past, global health targets have shifted the focus of nutrition surveillance from undernutrition to all forms of malnutrition, which also encompasses overweight and obesity [1, 2]. The fourth WHO Global Target recommends that countries should achieve no increase in childhood overweight by 2025. Also, the Sustainable Development Target 2.2 is aimed at ending all forms of malnutrition (Indicator 2.2.2 on wasting and overweight) among children under 5 years of age [3]. So far, however, the Global Nutrition Report states that no country is on target to achieve these targets [4].

Overweight and obesity are considered as a global epidemic by the World Health Organization (WHO), due to their rapid increase in several countries. In 2019, it is estimated that 38.2 million (5.6%) children under the age of 5 years were overweight or obese worldwide [5], with particularly high prevalence in upper-middle-income and high-income countries [6]. The worldwide increase is related to the nutritional transition, including urbanization, economic growth, and globalization, leading to lifestyle changes including reduced physical activity and poor dietary habits with increased intake of highly processed, high-energy foods [7, 8]. Whereas it is well known that prevalence of overweight increases with age after the age of 5 years [9], we were unable to find any studies showing age patterns in overweight prevalence in children aged under 5 years using data from multiple countries. Our expectation was to find an increase with age within this group.

In terms of undernutrition, 47 million children worldwide, or 6.9% of all under 5 children, suffered from wasting as of 2019 [10], and along with other forms of undernutrition accounts for 1 million child deaths annually [11]. Wasting is particularly common in South Asia, where the largest number of wasted children in the world are located [12]. Wasting, as other nutritional indicators, is closely linked to food insecurity, poor dietary quality, and disease, being concentrated in low-income settings [11, 13]. Although wasting prevalence is classically defined as being most common in the second year of life [14], recent studies show that younger children are more likely to be wasted [12, 15].

Even though all countries are experiencing some level of malnutrition, it is evident that differences exist regarding sociodemographic characteristics [4]. For some countries, the situation is even more concerning, with two or more extreme malnutrition scenarios simultaneously, such as high levels of undernutrition (stunting and/or wasting) and overweight, implicating in the double burden of malnutrition (DBM). This phenomenon can refer to maternal and child indicators of malnutrition in individual, household, or populational levels [16], and affects mostly low- and middle-income countries (LMICs), particularly in south and east Asia and sub-Saharan Africa [17]. A study with 93 LMICs showed that, among children under the age of 5 years, the national prevalence of DBM at individual level ranges from 0.2 to 10.9%, and one-third of the countries show higher levels of DBM than would be expected by chance [18].

The Sustainable Development Goals (SDG) target 17.18 recommends disaggregation of data by income, sex, age, ethnicity, and by additional relevant characteristics in national contexts. Regarding age of the child, there is a substantial literature on inequality in stunting prevalence [19], but less attention has been given to how wasting prevalence varies with age, and even less attention to age patterns in overweight prevalence. The aim of the present study is to describe overweight and wasting prevalence among under 5 children according to age in years, in low-and middle-income countries based on data from national surveys.

## Methods

### Data sources and measurements

The present analyses are based on Demographic and Health Surveys (DHS) [20] and Multiple Indicator Cluster Surveys (MICS) [21]. These nationally representative surveys collect data on reproductive, maternal, neonatal and child health and nutrition in LMICs. DHS and MICS are cross-sectional surveys which employ similar sampling methods, using multistage cluster procedures to select women of reproductive age (15–49 years) and children under 5 years of age. Both surveys use standardized questionnaires administered via face-to-face interviews by trained fieldworkers, permitting higher comparability between the surveys.

Publicly available data from DHS and MICS have been harmonized and reanalyzed by the International Center for Equity in Health (ICEH; www.equidade.org) to provide disaggregated estimates that enable the study of social and economic inequalities. The ICEH database currently comprises 412 surveys from 117 countries. The present analyses included DHS or MICS carried out from 2010 to 2019, in which anthropometric data were available for children under 5 years of age. For countries with more than one survey since 2010, the most recent one was analyzed.

Children up to 23 months had their supine length measured, while for children aged 24–59 months the standing height was obtained. For simplicity, we refer to weight for height (WHZ) to represent both weight for length and weight for height. The measuring boards used to collect length/height include ShorrBoards (Weight and Measure, LLC, Olney, MD, USA), SECA (Hamburg, Germany), as well as locally manufactured boards in some surveys. Children under 59 months of age were weighed using portable SECA 874 U electronic scales (Hamburg, Germany) [22, 23].

Overweight prevalence was defined as the proportion of children with mean WHZ more than 2 standard deviations above the median of the WHO reference curve [25, 26]. Wasting prevalence was defined as the proportion of children presenting mean WHZ more than −2 standard deviations below the median of the WHO reference curve. Both indicators were calculated based upon the 2006 WHO Child Growth Standards [25, 26]. Although the usual definition of *Z* scores is based on mean, rather than median values, the growth standards use the latter to account for the asymmetric distribution of weight [27–30]. According to the WHO recommendations, we considered WHZ individual scores between −5 and +5 of the WHO Growth Standards as valid measures [24].

Standard deviations that are markedly different from the expected value of 1.0 suggest the possibility of measurement error. Using the National Center for Health Statistics (USA) reference, the WHO suggested that standard deviation values outside the range of 0.85–1.1 for WHZ may indicate problems with anthropometric measurements [14, 24]. We assessed the variability of WHZ standard deviations by age and report on the number of countries with values outside 0.85–1.1.

We also analyzed overweight prevalence among children aged 2–5 years using the International Obesity Taskforce (IOTF) age- and sex-adjusted BMI cut-offs for overweight and obesity in children, which correspond to the adult BMI cut-offs (25 and 30 kg/m^−2^) [31]. The IOTF reference does not cover children aged under 2 years. Results using the IOTF BMI cut-offs for overweight are available as Supplementary Materials.

### Data analyses

Descriptive analyses include estimates of child overweight and wasting at national level, stratified by age in months (0–11, 12–23, 24–35, 36–47, 48–59). Ages were estimated by subtracting the birth date from the interview date. Logistic regression was used to test for within-country linear trends according to the child’s age in full years, by coding each child as overweight or not overweight, and in a separate regression as wasted or not wasted.

Comparisons among the five age groups were based on Chi-square tests, providing heterogeneity *p* values for the group differences on overweight and wasting prevalence across the five age groups. These analyses were performed separately for each outcome (overweight and wasting), analyzing all the countries included and each income group.

The graphic presentation of results included bar graphs for overweight and wasting prevalence by age and country income groups, and boxplots to show between-country variations in WHZ means and standard deviations. A scatter plot was used to relate mean WHZ to prevalence of overweight and wasting, using fractional polynomials to draw regression curves. All analyses were stratified by age groups and weighted by the under 5-year population in the median year of available surveys (2014). In the supplementary analyses, we used local polynomial smooth plots with confidence intervals (lpolyci command in Stata) to show mean levels by continuous age, also with national under five populations as weights.

All country-level analyses accounted for the multistage survey design, including sampling weights and clustering. Databases were handled using Stata 16.0 (StataCorp, College Station, TX, USA) and Microsoft Excel^®^ spreadsheets (Microsoft Corp, WA, USA). Ethical clearance for the national surveys was obtained at the country level by the research institutions involved in data collection.

## Results

The present analyses included 90 national surveys from LMIC’s carried out between 2010 and 2019 (median year = 2014). Countries included in the analyses represented 87.1% of all low-income countries in the world as of 2014, 70.6% of all lower-middle and 50.9% of all upper-middle-income countries. Supplementary Table 1 shows the numbers of children in each income group for all countries included in the analyses.

The median number of children under the age of 5 years with valid anthropometric and age information across all countries was 5920 (IQR = 3263; 10263). The median numbers by age group were as follows: 0–11 months (*N* = 1101; IQR = 538; 2100), 12–23 months (*N* = 1137; IQR = 624; 2080), 24–35 months (*N* = 1151; IQR = 653; 2071), 36–47 months (*N* = 1192; IQR = 612; 2125), and 48–59 months (*N* = 1079; IQR = 664; 1997).

**Fig. 1:**
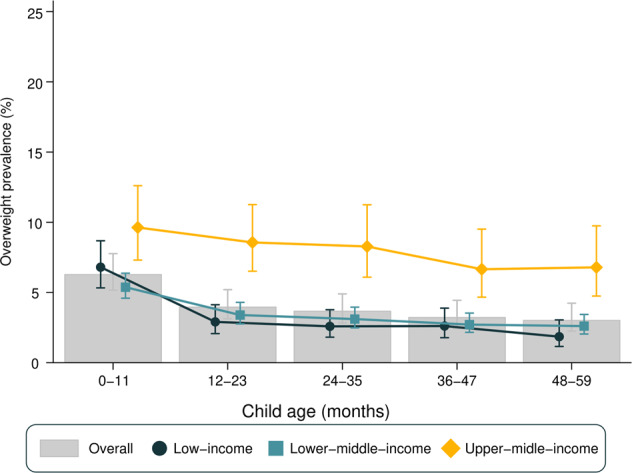
Overweight prevalence by age in all 90 LMIC (bars) and stratified by country income groups (symbols). Error bars represent 95% confidence intervals for the prevalence in each group.

Figure 1 presents overweight prevalence stratified by age and country income groups. Overall, the prevalence of overweight declined with increasing age (heterogeneity *p* value = 0.03), with a 3.3 percentage points (p.p.) of difference between the 0–11 (6.3%) and the 48–59 months (3.0%) age groups. A similar pattern was observed when stratifying for gender (Supplementary Fig. 1) and using the IOTF classification for overweight (Supplementary Fig. 4).

Overweight prevalence was considerably lower in low-income countries in comparison with upper-middle-income countries regardless of age group (Fig. 1). In low-income countries, overweight prevalence was at least 3.8 p.p. higher in the 0–11 months group than in older age groups (heterogeneity *p* value < 0.001). In lower-middle-income countries, this difference was at least 2.0 p.p., with a similar decline with age (heterogeneity *p* value < 0.001). As for upper-middle-income countries, there was an apparent decline in prevalence with age, but it was not as pronounced as in low-income or lower-middle-income countries and did not reach statistical significance (heterogeneity *p* value = 0.082).

Supplementary Table 2 presents the national prevalence of overweight stratified by age and respective SII values. Overweight prevalence among children aged 0–11 months varied from 1.8% in Mauritania to 23.3% in South Africa, whereas for children aged 48–59 months age the range was from 0.1% in Madagascar to 14.5% in Egypt. Tests for linear trends in overweight prevalence showed statistically significant declines with age in 65 countries and increases with age in only three countries: El Salvador, North Macedonia, and Thailand. For the remaining 22 countries, there was no evidence of a linear trend (Supplementary Table 2). As for national estimates, Senegal was the country with the lowest national overweight prevalence among all under 5 children (0.9%), while the highest prevalence was observed in Bosnia and Herzegovina (18.1%) (data not shown).

**Fig. 2:**
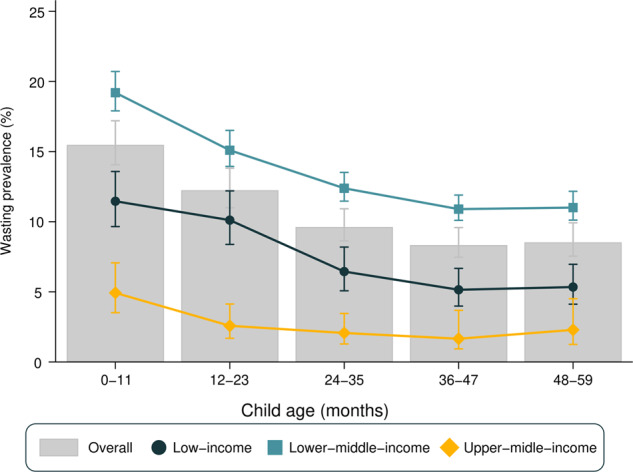
Wasting prevalence by age in all 90 LMIC (bars) and stratified by country income groups (symbols). Error bars represent 95% confidence intervals for the prevalence in each group.

Figure 2 presents wasting prevalence by age and country income groups. Wasting was more frequent among infants (0–11 months), with a slight decrease between the first and second year of life, and little variation thereafter. The same pattern was observed when stratifying for gender (Supplementary Fig. 3). When stratifying for country income groups, upper-middle-income countries presented the smallest prevalence, varying from 4.9% among infants to 1.7% among 3 years olds. Lower-middle-income countries showed the highest prevalence in all age groups (ranging from 19.0% among infants to 10.9% among 3 years olds). In low-income countries, prevalence ranged from 11.5% in infants to 5.2% in 3 years olds. All groups of countries showed a decline with age from infants to 3 years olds, with stability thereafter.

Wasting prevalence among children aged 0–11 months varied from 0.8% in Peru to 28.9% in India, whereas for children aged 48–59 months age the range was from 0.0% in Eswatini to 22.5% in Timor-Leste. Tests for linear trends in wasting prevalence showed statistically significant declines with age in 72 countries and increases with age only in the Maldives. For the remaining 17 countries, there was no evidence of a linear trend (Supplementary Table 3). Regarding national estimates, Peru showed the lowest national wasting prevalence (0.5%) and Timor-Leste the highest (24.0%) (data not shown).

As a consequence of the higher prevalence of both overweight and wasting among younger children, the proportions of children with appropriate WHZ (between −2 and +2 *Z* scores) increased with age, being approximately to 78, 85, 88, 89, and 90% for age groups 0–11, 12–23, 24–35, 36–47, and 48–59 months, respectively (Supplementary Fig. 6).

**Fig. 3:**
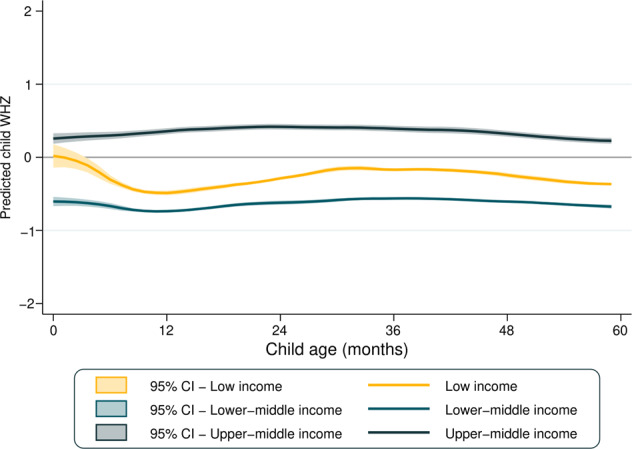
The lines represent mean WHZ according to age in each country income group, while the shades represent 95% confidence intervals.

Figure 3 presents the mean WHZ according to country income groups. Regardless of age, upper-middle-income countries showed the highest mean WHZ, which was a little above 0 for all ages. Mean WHZ was stable in lower-middle and upper-middle-income countries, while for low-income countries a decline is observed between 0 and 12 months of age.

In Supplementary Fig. 4, the boxplots show the distribution in all countries studied of WHZ scores and respective standard deviations across age groups. Median values of the national-level WHZ means were close to zero, being similar among all age groups (heterogeneity *p* value = 0.659), median of the standard deviations of the national-level WHZ means were higher among infants than older children (heterogeneity *p* value < 0.001), for whom standard deviations were just above 1.0. Out of the 90 surveys included in the present analyses, in 86 the standard deviation for infants exceeded the maximum recommended value of 1.1; the corresponding numbers of surveys exceeding this cutoff were 71, 49, 44, and 41 for children aged 1–4 years, respectively.

**Fig. 4:**
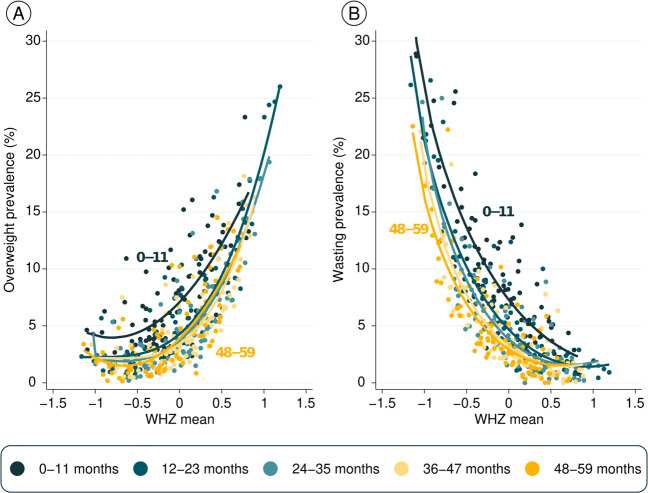
Mean prevalence of overweight and wasting according to mean WHZ, by age (*N* = 450). Comparison between mean WHZ and prevalence of overweight (**A**) and wasting (**B**). Each dot represents one age group in a country, totaling 450 combinations of country and age group.

Figure 4 shows prevalence of the two outcomes (overweight and wasting) according to mean WHZ by age. Each dot represents one age group in a country, totaling 450 observations. The fitted curves for infants are well above the curves for older children, showing that the same level of mean WHZ is associated both with higher overweight (Fig. 4A) as well as higher wasting prevalence (Fig. 4B) in infants than for older children. This pattern is due to the wider standard deviations among infants, as shown in Supplementary Fig. 4B.

## Discussion

We report on patterns of child overweight and wasting prevalence in 90 LMICs, according to age in years. We are unaware of any previous publications on this issue including data from a large number of countries.

In accordance with the literature, we found that overweight prevalence showed a gradual reduction from upper-middle-income to low-income countries, with intermediate prevalence in lower-middle-income countries [32]. In contrast, wasting prevalence was higher in lower-middle-income countries than in low-income countries, an apparently paradoxical finding that is largely driven by high prevalence in the populous South Asian countries which are classified in the lower-middle-income group [12]. Wasting prevalence, as expected, was very low in upper-middle-income countries.

Regarding age patterns, we found that prevalence of both overweight and wasting declined with age. The proportions of children classified in the normal range for WHZ increased from 78% in infants to 90% in 4-year-old children, and therefore the combined prevalence of wasting and overweight ranged from 22% in infants to 10% in 4-year-old children. The latter values correspond to SDG indicator 2.2.2, which defines malnutrition as weight for height > +2 or <−2 standard deviations from the median of the WHO Child Growth Standards.

Our original hypothesis was that overweight prevalence would increase with age among under 5 children, mainly due to breastfeeding cessation and the increasing consumption of high-energy foods [33]. Yet, our results showed a decline in overweight prevalence with age in all three country income groups, and in most countries studied. This observation led us to investigate how mean WHZ varied with age, but no clear pattern was observed. The anthropometry literature suggests that use of mean *Z* scores in child anthropometry has the advantage of describing the nutritional status of the entire population directly, without resorting to a subset of individuals below or above a set cutoff [14]. In addition, mean values are less likely to be affected by outliers than estimates based on the tails of the distribution.

In light of the contrasting findings of higher prevalence in young children side by side with constant mean WHZ, we investigated how the standard deviation of WHZ measures varied with age. These analyses revealed that standard deviations were markedly higher for infants—and to a lesser extent among 1 year olds—than for older children. A possible explanation is that length or height is more difficult to measure reliably in younger children [34, 35], for whom an error of a few centimeters could markedly affect the WHZ value. For example, an error of only 1.5 cm in length or height measurement could result in ~0.1 in the WHZ mean and 3 percent points in wasting prevalence [36]. Earlier evaluations of DHS and MICS surveys showed heterogeneity in anthropometric data quality between and within surveys and over time, although there is evidence of improvements in more recent surveys such as those included in the present analyses [37, 38]. We are unaware of any previous multicountry analyses examining how the standard deviations of WHZ vary with age in existing surveys.

To further assess the hypothesis of measurement error, we compared the mean WHZ with overweight and wasting prevalence in the same survey. Among infants, a given level of mean WHZ was associated with substantially higher prevalence of both overweight and wasting than among older children.

In addition to measurement quality issues, another possible explanation for the wider standard deviations observed among young children is that they are more likely to cross growth percentiles, whereas children aged 2 years or more tend to grow along the same percentile, which is known as “growth canalization.” Rapid crossing of growth channels in young children could result in cross-sectional findings of high prevalence of both overweight and wasting compared to older children whose growth channels are more stable [39–41].

Our study presents some limitations. First, the percentage of countries with available surveys is not equally distributed according to World Bank income groups, as surveys were available for 87.1% of low-income, 70.6% of lower-middle-income, and 50.9% of upper-middle-income countries. Second, we included surveys from 2010 onward (median year = 2014), and for countries without recent surveys national estimates may be outdated.

In conclusion, our study showed a pattern of decline in overweight and wasting prevalence with age in under 5 children, whereas mean WHZ values were constant. The decline with age is explained by wider standard deviations among young children which may be due to measurement error or by rapid crossing of growth channels. While these issues remain, it is not possible to make any firm statements about variability in prevalence of overweight by age on the basis of routinely carried out anthropometric surveys. Cohort and cross-sectional studies with high-quality anthropometry are needed to assess how overweight and wasting vary with the ages of young children.

## Supplementary information

Supplementary material
