# Supplementary material for: Age patterns in overweight and wasting prevalence of under 5 years-old children from low- and middle-income countries
Source: Int J Obes (Lond). Author manuscript; Available in PMC 2021 Oct 25. (PMC8528694; doi:10.1038/s41366-021-00911-5)
Supplement: Supplementary material [file EMS129586-supplement-Supplementary_material.docx]

**Supplementary material**

**Supplementary Figure 1:** Overweight prevalence stratified by age groups (months) and sex in low- and middle- income countries (N=90)

**Supplementary Figure 2:** Wasting prevalence stratified by age groups (months) and sex in low- and middle- income countries (N=90)

**Supplementary Figure 3.** Overweight prevalence (IOTF standard) stratified by age groups (months) in LMIC’s (N= 90)

**Supplementary table 1: Number of children with valid WHZ information by country income and age group**

|  | Number of children | | | | | |
| --- | --- | --- | --- | --- | --- | --- |
| Country income group | Overall | 0-11 | 12-23 | 24-35 | 36-47 | 48-59 |
| Low-income countries | 204710 | 41183 | 41164 | 40268 | 40761 | 41334 |
| Lower-middle-income countries | 471320 | 87767 | 93411 | 93565 | 97825 | 98752 |
| Upper-middle-income countries | 146237 | 26232 | 28983 | 29762 | 30531 | 30729 |

| **Supplementary table 2: National prevalence of overweight stratified by age** | | | | | |  |  |  |  |
| --- | --- | --- | --- | --- | --- | --- | --- | --- | --- |
| **Survey** | **Source** | **N** | **Weight-for-height (>2 SD)** | | | | | | |
|  |  |  | **0-11** | **12-23** | **24-35** | **36-47** | **48-59** | **OR** | **95% CI** |
| Low-income countries | | | | | | | | | |
| Benin (2017) | DHS | 13240 | 4.0 | 1.2 | 2.1 | 1.3 | 0.7 | **0.71** | **0.64; 0.79** |
| Burkina Faso (2010) | DHS | 6888 | 2.9 | 1.9 | 3.2 | 3.1 | 2.8 | 1.06 | 0.96; 1.17 |
| Burundi (2016) | DHS | 6367 | 2.3 | 1.1 | 1.5 | 1.4 | 0.6 | **0.73** | **0.63; 0.85** |
| Cambodia (2014) | DHS | 4897 | 5.5 | 2.2 | 2.0 | 0.9 | 0.9 | **0.84** | **0.75; 0.95** |
| Central African Republic (2010) | MICS | 10263 | 3.3 | 0.8 | 1.3 | 2.0 | 1.5 | **0.66** | **0.57; 0.78** |
| Chad (2014) | DHS | 11086 | 2.7 | 1.8 | 3.4 | 3.3 | 2.8 | 1.02 | 0.94; 1.10 |
| Comoros (2012) | DHS | 2785 | 9.8 | 10.4 | 13.1 | 8.6 | 10.3 | 1.03 | 0.95; 1.13 |
| Congo Democratic Republic (2017) | MICS | 21079 | 7.8 | 2.9 | 2.8 | 2.7 | 2.8 | **0.82** | **0.78; 0.86** |
| Ethiopia (2016) | DHS | 9616 | 6.7 | 2.6 | 1.5 | 2.2 | 1.2 | **0.69** | **0.61; 0.77** |
| Gambia (2018) | MICS | 9711 | 3.7 | 1.2 | 0.6 | 0.5 | 0.4 | **0.56** | **0.47; 0.68** |
| Guinea (2018) | DHS | 3996 | 10.1 | 4.2 | 6.9 | 4.7 | 4.3 | **0.79** | **0.71; 0.88** |
| Guinea Bissau (2014) | MICS | 7515 | 6.2 | 1.5 | 1.1 | 1.8 | 1.1 | **0.63** | **0.55; 0.72** |
| Haiti (2016) | DHS | 6740 | 6.1 | 2.5 | 2.9 | 3.6 | 2.4 | **0.80** | **0.72; 0.89** |
| Liberia (2013) | DHS | 3857 | 5.0 | 3.0 | 1.5 | 2.4 | 4.0 | **0.82** | **0.71; 0.95** |
| Madagascar (2018) | MICS | 12437 | 5.0 | 0.6 | 0.5 | 0.5 | 0.1 | **0.35** | **0.28; 0.44** |
| Malawi (2015) | DHS | 5734 | 11.2 | 3.5 | 2.5 | 3.2 | 2.1 | **0.63** | **0.56; 0.71** |
| Mali (2018) | DHS | 9496 | 3.3 | 1.2 | 1.7 | 1.8 | 1.7 | 0.94 | 0.85; 1.04 |
| Mozambique (2011) | DHS | 10501 | 13.0 | 7.7 | 8.1 | 5.6 | 4.7 | **0.76** | **0.72; 0.80** |
| Nepal (2016) | DHS | 2442 | 3.7 | 0.9 | 0.2 | 0.5 | 0.9 | **0.62** | **0.43; 0.90** |
| Niger (2012) | DHS | 5331 | 6.3 | 2.3 | 3.0 | 1.7 | 1.6 | **0.74** | **0.66; 0.83** |
| Rwanda (2014) | DHS | 3807 | 13.8 | 8.5 | 7.3 | 6.4 | 3.2 | **0.72** | **0.66; 0.79** |
| Senegal (2017) | DHS | 12071 | 2.8 | 1.0 | 0.3 | 0.4 | 0.2 | **0.43** | **0.33; 0.56** |
| Sierra Leone (2017) | MICS | 11435 | 9.1 | 2.2 | 3.9 | 4.3 | 2.0 | **0.76** | **0.71; 0.81** |
| Tajikistan (2017) | DHS | 6019 | 4.0 | 2.7 | 3.4 | 4.3 | 2.1 | 0.91 | 0.83; 1.00 |
| Tanzania (2015) | DHS | 10153 | 8.1 | 3.2 | 2.6 | 3.1 | 1.0 | **0.65** | **0.59; 0.71** |
| Togo (2017) | MICS | 4906 | 4.1 | 0.7 | 1.2 | 0.8 | 0.7 | **0.62** | **0.50; 0.79** |
| Uganda (2016) | DHS | 5223 | 7.4 | 4.9 | 2.3 | 3.1 | 1.7 | **0.74** | **0.65; 0.83** |
| Lower-middle-income countries | | | | | | | | | |
| Armenia (2015) | DHS | 1575 | 11.9 | 17.9 | 12.0 | 14.0 | 11.9 | 0.93 | 0.84; 1.04 |
| Bangladesh (2019) | MICS | 22053 | 3.7 | 2.7 | 2.4 | 2.0 | 1.4 | **0.78** | **0.72; 0.83** |
| Bhutan (2010) | MICS | 5845 | 11.7 | 8.3 | 6.8 | 6.7 | 4.8 | **0.87** | **0.81; 0.94** |
| Cameroon (2018) | DHS | 5135 | 14.7 | 9.5 | 10.9 | 10.8 | 8.7 | **0.89** | **0.83; 0.95** |
| Congo Brazzaville (2014) | MICS | 8699 | 7.9 | 6.4 | 6.2 | 4.4 | 4.6 | **0.90** | **0.83; 0.97** |
| Cote dIvoire (2016) | MICS | 8958 | 2.4 | 1.4 | 0.9 | 1.5 | 1.1 | **0.85** | **0.75; 0.97** |
| Egypt (2014) | DHS | 14265 | 16.1 | 16.3 | 16.8 | 14.6 | 14.5 | **0.91** | **0.88; 0.94** |
| El Salvador (2014) | MICS | 7194 | 6.3 | 5.7 | 6.3 | 6.4 | 7.1 | **1.08** | **1.00; 1.15** |
| Eswatini (2014) | MICS | 2635 | 17.4 | 8.8 | 7.3 | 7.7 | 4.5 | **0.71** | **0.63; 0.79** |
| Ghana (2017) | MICS | 8766 | 2.5 | 0.5 | 1.7 | 1.2 | 1.0 | 0.88 | 0.76; 1.02 |
| Guatemala (2014) | DHS | 12268 | 12.7 | 2.3 | 3.0 | 3.2 | 3.7 | **0.68** | **0.63; 0.73** |
| Guyana (2014) | MICS | 2972 | 3.9 | 5.7 | 9.0 | 6.1 | 1.7 | **0.90** | **0.81; 0.99** |
| Honduras (2011) | DHS | 10937 | 7.4 | 4.4 | 4.5 | 5.4 | 4.1 | **0.84** | **0.79; 0.90** |
| India (2015) | DHS | 236707 | 4.6 | 2.4 | 1.7 | 1.7 | 1.9 | **0.73** | **0.72; 0.75** |
| Kenya (2014) | DHS | 20707 | 9.7 | 4.2 | 2.8 | 2.8 | 1.9 | **0.61** | **0.57; 0.65** |
| Kiribati (2018) | MICS | 2142 | 3.4 | 2.6 | 1.1 | 2.4 | 0.8 | **0.76** | **0.60; 0.96** |
| Kosovo (2013) | MICS | 1505 | 2.9 | 5.3 | 4.7 | 4.4 | 4.3 | 1.06 | 0.89; 1.25 |
| Kyrgyzstan (2018) | MICS | 3430 | 9.9 | 7.9 | 7.2 | 4.6 | 5.1 | **0.78** | **0.70; 0.86** |
| Lao (2017) | MICS | 11333 | 5.4 | 3.5 | 3.2 | 2.6 | 3.0 | **0.80** | **0.74; 0.87** |
| Lesotho (2018) | MICS | 3148 | 13.2 | 6.7 | 5.4 | 4.2 | 4.9 | **0.75** | **0.67; 0.84** |
| Mauritania (2015) | MICS | 10054 | 1.8 | 1.1 | 1.4 | 1.4 | 0.7 | 0.89 | 0.79; 1.01 |
| Moldova (2012) | MICS | 1660 | 5.8 | 7.8 | 4.1 | 1.8 | 4.4 | 0.84 | 0.71; 1.00 |
| Mongolia (2018) | MICS | 5894 | 12.0 | 13.9 | 10.5 | 10.8 | 5.7 | **0.80** | **0.75; 0.84** |
| Myanmar (2015) | DHS | 4617 | 3.1 | 1.6 | 0.6 | 1.1 | 1.3 | **0.62** | **0.50; 0.76** |
| Nigeria (2018) | DHS | 12430 | 3.8 | 1.6 | 1.8 | 1.6 | 1.2 | **0.73** | **0.66; 0.82** |
| Pakistan (2017) | DHS | 3518 | 3.8 | 2.3 | 3.7 | 1.3 | 1.5 | **0.79** | **0.69; 0.89** |
| Papua New Guinea (2016) | DHS | 4028 | 15.2 | 9.3 | 7.5 | 7.5 | 7.1 | **0.85** | **0.77; 0.94** |
| Sao Tome and Principe (2014) | MICS | 1946 | 3.9 | 2.1 | 3.1 | 2.0 | 1.2 | 0.84 | 0.68; 1.03 |
| South Sudan (2010) | MICS | 5947 | 7.1 | 5.4 | 5.3 | 7.5 | 4.8 | 0.99 | 0.91; 1.08 |
| State of Palestine (2014) | MICS | 6896 | 10.4 | 9.2 | 9.1 | 6.6 | 5.8 | **0.85** | **0.80; 0.90** |
| Sudan (2014) | MICS | 12402 | 5.1 | 1.7 | 2.3 | 3.0 | 2.7 | **0.79** | **0.72; 0.86** |
| Timor Leste (2016) | DHS | 6436 | 10.9 | 5.7 | 4.3 | 3.4 | 3.4 | **0.76** | **0.69; 0.83** |
| Tunisia (2018) | MICS | 3263 | 12.4 | 26.0 | 19.4 | 15.0 | 13.1 | **0.91** | **0.86; 0.97** |
| Vietnam (2010) | MICS | 3558 | 3.2 | 6.3 | 4.7 | 3.4 | 4.3 | 1.01 | 0.91; 1.13 |
| Yemen (2013) | DHS | 14269 | 4.4 | 2.4 | 1.7 | 1.9 | 1.8 | **0.80** | **0.74; 0.87** |
| Zambia (2018) | DHS | 9584 | 10.4 | 5.3 | 4.2 | 3.6 | 2.3 | **0.68** | **0.63; 0.74** |
| Zimbabwe (2019) | MICS | 5959 | 5.7 | 1.4 | 2.7 | 2.2 | 0.9 | **0.68** | **0.60; 0.78** |
| Upper-middle-income countries | | | | | | | | | |
| Albania (2017) | DHS | 2580 | 15.7 | 24.4 | 14.4 | 16.3 | 13.3 | **0.88** | **0.82; 0.95** |
| Algeria (2012) | MICS | 13635 | 13.8 | 17.8 | 11.3 | 8.9 | 9.0 | **0.84** | **0.81; 0.87** |
| Angola (2015) | DHS | 7646 | 7.5 | 2.3 | 3.5 | 3.0 | 1.3 | **0.70** | **0.63; 0.78** |
| Belize (2015) | MICS | 2402 | 7.6 | 6.9 | 8.5 | 5.7 | 8.0 | 0.90 | 0.81; 1.01 |
| Bosnia and Herzegovina (2011) | MICS | 2093 | 12.3 | 24.7 | 17.9 | 18.2 | 13.5 | **0.90** | **0.83; 0.98** |
| Colombia (2010) | DHS | 17804 | 6.0 | 5.5 | 3.7 | 5.0 | 4.1 | **0.88** | **0.84; 0.93** |
| Dominican Republic (2013) | DHS | 3666 | 10.9 | 10.1 | 8.6 | 4.4 | 4.9 | **0.78** | **0.71; 0.85** |
| Gabon (2012) | DHS | 4115 | 11.5 | 9.0 | 8.4 | 3.7 | 6.0 | **0.81** | **0.74; 0.89** |
| Georgia (2018) | MICS | 2002 | 2.4 | 8.3 | 7.4 | 6.7 | 5.3 | 1.01 | 0.91; 1.13 |
| Iraq (2018) | MICS | 16295 | 7.3 | 8.5 | 6.0 | 5.2 | 4.1 | **0.89** | **0.85; 0.93** |
| Jordan (2012) | DHS | 6394 | 9.1 | 5.6 | 3.7 | 2.6 | 3.6 | **0.80** | **0.74; 0.87** |
| Kazakhstan (2015) | MICS | 5189 | 9.4 | 12.7 | 10.5 | 8.0 | 6.5 | **0.87** | **0.82; 0.93** |
| Maldives (2016) | DHS | 2511 | 4.7 | 4.3 | 3.2 | 3.3 | 5.6 | 0.99 | 0.85; 1.15 |
| Mexico (2015) | MICS | 7831 | 6.0 | 2.5 | 4.9 | 5.7 | 6.6 | 1.04 | 0.96; 1.12 |
| Montenegro (2018) | MICS | 773 | 6.2 | 8.8 | 5.6 | 9.0 | 6.8 | 0.96 | 0.83; 1.11 |
| Namibia (2013) | DHS | 2608 | 6.5 | 4.5 | 4.4 | 2.5 | 2.0 | **0.72** | **0.62; 0.83** |
| North Macedonia (2011) | MICS | 1297 | 3.3 | 12.8 | 17.9 | 15.6 | 11.8 | **1.20** | **1.08; 1.33** |
| Paraguay (2016) | MICS | 4407 | 11.9 | 14.1 | 13.1 | 12.9 | 10.2 | 0.99 | 0.93; 1.05 |
| Peru (2018) | DHS | 23278 | 13.3 | 6.5 | 5.9 | 8.4 | 9.9 | **0.95** | **0.91; 0.98** |
| Serbia (2014) | MICS | 2363 | 8.2 | 23.3 | 15.9 | 12.0 | 11.3 | **0.92** | **0.85; 0.99** |
| South Africa (2016) | DHS | 1449 | 23.3 | 11.6 | 14.4 | 9.8 | 10.1 | **0.76** | **0.67; 0.87** |
| St Lucia (2012) | MICS | 275 | 9.3 | 6.1 | 1.5 | 4.1 | 11.0 | 0.89 | 0.59; 1.32 |
| Suriname (2018) | MICS | 3378 | 3.0 | 5.8 | 3.1 | 1.9 | 4.0 | 1.02 | 0.90; 1.16 |
| Thailand (2015) | MICS | 11138 | 3.2 | 6.4 | 10.9 | 9.3 | 10.2 | **1.08** | **1.03; 1.13** |
| Turkey (2013) | DHS | 2774 | 11.1 | 15.3 | 12.5 | 6.7 | 9.2 | **0.85** | **0.78; 0.93** |
| Turkmenistan (2015) | MICS | 3708 | 5.5 | 5.9 | 4.7 | 7.7 | 5.6 | 1.08 | 0.99; 1.18 |

| **Supplementary table 3: National prevalence of wasting stratified by age** | | | | | | | | | |
| --- | --- | --- | --- | --- | --- | --- | --- | --- | --- |
| **Survey** | **Source** | **N** | **Weight-for-height (<2 SD)** | | | | |  |  |
|  |  |  | **0-11** | **12-23** | **24-35** | **36-47** | **48-59** | **OR** | **95% CI** |
| Low-income countries | | | | | | | | | |
| Benin (2017) | DHS | 13240 | 8.0 | 7.2 | 3.4 | 2.1 | 3.4 | **0.72** | **0.68; 0.77** |
| Burkina Faso (2010) | DHS | 6888 | 28.7 | 21.5 | 12.6 | 8.2 | 6.5 | **0.64** | **0.61; 0.67** |
| Burundi (2016) | DHS | 6367 | 7.2 | 8.0 | 4.0 | 2.2 | 3.5 | **0.74** | **0.68; 0.81** |
| CAR (2010) | MICS | 10263 | 9.0 | 13.3 | 6.3 | 3.1 | 4.4 | **0.76** | **0.72; 0.80** |
| Cambodia (2014) | DHS | 4897 | 12.0 | 11.0 | 8.2 | 9.3 | 8.8 | **0.90** | **0.84; 0.96** |
| Chad (2014) | DHS | 11086 | 17.2 | 19.5 | 12.0 | 9.8 | 9.1 | **0.83** | **0.80; 0.86** |
| Comoros (2012) | DHS | 2785 | 18.4 | 12.4 | 6.7 | 8.6 | 10.2 | **0.82** | **0.75; 0.90** |
| Congo Democratic Republic (2017) | MICS | 21079 | 8.2 | 8.4 | 5.3 | 5.0 | 5.3 | **0.84** | **0.81; 0.87** |
| Ethiopia (2016) | DHS | 9616 | 14.5 | 12.7 | 9.4 | 6.9 | 6.7 | **0.84** | **0.80; 0.88** |
| Gambia (2018) | MICS | 9711 | 6.6 | 6.9 | 4.2 | 5.9 | 7.3 | 0.95 | 0.89; 1.01 |
| Guinea (2018) | DHS | 3996 | 11.8 | 9.0 | 10.2 | 6.9 | 7.6 | **0.86** | **0.79; 0.94** |
| Guinea Bissau (2014) | MICS | 7515 | 6.8 | 7.7 | 5.6 | 3.7 | 5.9 | **0.88** | **0.82; 0.94** |
| Haiti (2016) | DHS | 6740 | 8.1 | 4.4 | 2.8 | 1.6 | 1.7 | **0.65** | **0.58; 0.74** |
| Liberia (2013) | DHS | 3857 | 10.5 | 7.5 | 4.7 | 2.2 | 2.6 | **0.64** | **0.58; 0.71** |
| Madagascar (2018) | MICS | 12437 | 7.1 | 8.2 | 4.7 | 6.0 | 5.9 | **0.94** | **0.89; 0.99** |
| Malawi (2015) | DHS | 5734 | 4.0 | 3.4 | 2.4 | 1.8 | 2.6 | **0.81** | **0.72; 0.91** |
| Mali (2018) | DHS | 9496 | 12.7 | 13.3 | 6.1 | 5.4 | 5.9 | **0.75** | **0.71; 0.80** |
| Mozambique (2011) | DHS | 10501 | 10.7 | 8.7 | 5.6 | 2.1 | 3.1 | **0.68** | **0.63; 0.72** |
| Nepal (2016) | DHS | 2442 | 17.0 | 12.4 | 6.8 | 6.3 | 6.6 | **0.76** | **0.68; 0.84** |
| Niger (2012) | DHS | 5331 | 24.8 | 26.2 | 15.7 | 12.7 | 12.6 | **0.81** | **0.77; 0.85** |
| Rwanda (2014) | DHS | 3807 | 5.1 | 3.2 | 1.4 | 0.6 | 1.2 | **0.60** | **0.49; 0.72** |
| Senegal (2017) | DHS | 12071 | 9.2 | 10.8 | 7.6 | 8.4 | 8.9 | 0.97 | 0.93; 1.02 |
| Sierra Leone (2017) | MICS | 11435 | 9.5 | 12.0 | 6.3 | 3.7 | 2.9 | **0.68** | **0.65; 0.72** |
| Tajikistan (2017) | DHS | 6019 | 11.8 | 7.0 | 3.9 | 2.0 | 2.8 | **0.66** | **0.61; 0.72** |
| Tanzania (2015) | DHS | 10153 | 8.2 | 5.3 | 3.0 | 3.1 | 3.6 | **0.75** | **0.69; 0.81** |
| Togo (2017) | MICS | 4906 | 6.9 | 8.3 | 7.0 | 2.8 | 3.4 | **0.81** | **0.74; 0.88** |
| Uganda (2016) | DHS | 5223 | 8.5 | 4.1 | 2.0 | 1.6 | 1.6 | **0.62** | **0.54; 0.71** |
| Lower-middle-income countries | | | | | | | | | |
| Armenia (2015) | DHS | 1575 | 4.8 | 1.6 | 5.1 | 6.3 | 4.6 | 1.09 | 0.92; 1.28 |
| Bangladesh (2019) | MICS | 22053 | 9.9 | 10.8 | 9.1 | 8.4 | 10.9 | 0.99 | 0.96; 1.02 |
| Bhutan (2010) | MICS | 5845 | 12.3 | 6.2 | 3.3 | 4.9 | 3.3 | **0.73** | **0.66; 0.80** |
| Cameroon (2018) | DHS | 5135 | 6.6 | 5.2 | 2.5 | 3.7 | 3.9 | **0.86** | **0.76; 0.96** |
| Congo Brazzaville (2014) | MICS | 8699 | 12.5 | 10.0 | 6.7 | 6.2 | 5.4 | **0.76** | **0.71; 0.81** |
| Cote dIvoire (2016) | MICS | 8958 | 10.9 | 9.1 | 5.0 | 3.0 | 2.2 | **0.65** | **0.61; 0.70** |
| Egypt (2014) | DHS | 14265 | 13.9 | 10.0 | 8.9 | 7.8 | 5.9 | **0.83** | **0.80; 0.86** |
| El Salvador (2014) | MICS | 7194 | 4.1 | 2.1 | 1.5 | 1.3 | 1.8 | **0.77** | **0.67; 0.87** |
| Eswatini (2014) | MICS | 2635 | 4.4 | 2.7 | 2.0 | 1.0 | 0.0 | **0.54** | **0.43; 0.68** |
| Ghana (2017) | MICS | 8766 | 14.2 | 10.2 | 4.7 | 3.1 | 2.8 | **0.66** | **0.62; 0.71** |
| Guatemala (2014) | DHS | 12268 | 0.9 | 1.1 | 0.8 | 0.6 | 0.4 | **0.86** | **0.74; 0.99** |
| Guyana (2014) | MICS | 2972 | 7.5 | 5.3 | 5.9 | 5.1 | 8.5 | 1.00 | 0.89; 1.12 |
| Honduras (2011) | DHS | 10937 | 2.4 | 1.7 | 1.1 | 0.6 | 0.9 | **0.74** | **0.66; 0.85** |
| India (2015) | DHS | 236707 | 28.9 | 21.8 | 19.3 | 17.6 | 17.3 | **0.86** | **0.85; 0.87** |
| Kenya (2014) | DHS | 20707 | 5.5 | 5.3 | 3.1 | 3.4 | 3.8 | **0.95** | **0.91; 0.99** |
| Kiribati (2018) | MICS | 2142 | 8.5 | 3.9 | 2.6 | 1.8 | 0.3 | **0.52** | **0.42; 0.64** |
| Kosovo (2013) | MICS | 1505 | 3.0 | 1.4 | 1.1 | 0.7 | 0.7 | **0.62** | **0.44; 0.88** |
| Kyrgyzstan (2018) | MICS | 3430 | 4.1 | 2.0 | 2.1 | 1.3 | 0.6 | **0.67** | **0.56; 0.82** |
| Lao (2017) | MICS | 11333 | 10.8 | 9.5 | 8.2 | 8.3 | 8.5 | **0.93** | **0.89; 0.98** |
| Lesotho (2018) | MICS | 3148 | 3.7 | 2.3 | 1.3 | 1.7 | 1.8 | 0.83 | 0.69; 1.01 |
| Mauritania (2015) | MICS | 10054 | 18.9 | 17.1 | 13.7 | 12.2 | 12.9 | **0.88** | **0.84; 0.91** |
| Moldova (2012) | MICS | 1660 | 3.1 | 1.6 | 1.9 | 2.2 | 0.5 | 0.80 | 0.62; 1.03 |
| Mongolia (2018) | MICS | 5894 | 2.2 | 0.7 | 0.4 | 0.5 | 0.8 | **0.79** | **0.65; 0.95** |
| Myanmar (2015) | DHS | 4617 | 8.9 | 8.1 | 5.0 | 5.0 | 6.5 | **0.86** | **0.79; 0.94** |
| Nigeria (2018) | DHS | 12430 | 10.4 | 11.1 | 5.5 | 3.0 | 3.7 | **0.71** | **0.67; 0.75** |
| Pakistan (2017) | DHS | 3518 | 13.5 | 7.4 | 5.8 | 4.3 | 3.9 | **0.75** | **0.69; 0.82** |
| Papua New Guinea (2016) | DHS | 4028 | 12.8 | 12.0 | 9.0 | 6.6 | 6.9 | **0.79** | **0.72; 0.86** |
| Sao Tome and Principe (2014) | MICS | 1946 | 8.7 | 5.2 | 1.6 | 2.4 | 3.0 | **0.72** | **0.60; 0.88** |
| South Sudan (2010) | MICS | 5947 | 24.6 | 22.9 | 25.0 | 19.2 | 22.2 | **0.94** | **0.90; 0.99** |
| State of Palestine (2014) | MICS | 6896 | 2.3 | 1.1 | 1.1 | 0.7 | 0.8 | **0.76** | **0.64; 0.89** |
| Sudan (2014) | MICS | 12402 | 15.4 | 21.2 | 17.1 | 13.2 | 15.2 | **0.95** | **0.92; 0.98** |
| Timor-Leste (2016) | DHS | 6436 | 25.6 | 26.6 | 24.7 | 22.1 | 22.5 | **0.93** | **0.89; 0.98** |
| Tunisia (2018) | MICS | 3263 | 5.8 | 0.9 | 1.5 | 1.3 | 1.5 | **0.76** | **0.62; 0.94** |
| Vietnam (2010) | MICS | 3558 | 6.7 | 3.8 | 2.2 | 3.5 | 5.0 | 0.88 | 0.77; 1.00 |
| Yemen (2013) | DHS | 14269 | 22.6 | 19.9 | 14.0 | 12.9 | 12.3 | **0.83** | **0.80; 0.85** |
| Zambia (2018) | DHS | 9584 | 5.2 | 5.3 | 4.0 | 2.9 | 3.8 | **0.87** | **0.81; 0.94** |
| Zimbabwe (2019) | MICS | 5959 | 3.8 | 4.4 | 1.5 | 2.3 | 2.5 | **0.82** | **0.74; 0.92** |
| Upper-middle-income countries | | | | | | | | | |
| Albania (2017) | DHS | 2580 | 2.6 | 0.4 | 1.3 | 0.6 | 1.8 | 0.88 | 0.70; 1.09 |
| Algeria (2012) | MICS | 13635 | 8.7 | 2.9 | 3.1 | 2.8 | 2.3 | **0.70** | **0.65; 0.75** |
| Angola (2015) | DHS | 7646 | 6.9 | 6.8 | 4.9 | 2.2 | 3.9 | **0.80** | **0.73; 0.87** |
| Belize (2015) | MICS | 2402 | 3.3 | 1.1 | 1.7 | 1.5 | 1.3 | 0.81 | 0.63; 1.03 |
| Bosnia and Herzegovina (2011) | MICS | 2093 | 5.4 | 1.2 | 1.4 | 1.8 | 2.2 | **0.71** | **0.54; 0.94** |
| Colombia (2010) | DHS | 17804 | 1.7 | 0.9 | 0.8 | 0.5 | 0.7 | **0.77** | **0.69; 0.86** |
| Dominican Republic (2013) | DHS | 3666 | 4.1 | 1.6 | 2.1 | 2.3 | 1.9 | 0.93 | 0.80; 1.09 |
| Gabon (2012) | DHS | 4115 | 5.8 | 2.4 | 3.2 | 2.6 | 3.2 | **0.75** | **0.66; 0.85** |
| Georgia (2018) | MICS | 2002 | 2.6 | 0.3 | 0.4 | 0.0 | 0.0 | **0.50** | **0.26; 0.94** |
| Iraq (2018) | MICS | 16295 | 7.4 | 2.5 | 2.0 | 1.8 | 1.8 | **0.66** | **0.61; 0.71** |
| Jordan (2012) | DHS | 6394 | 3.5 | 1.8 | 1.5 | 2.7 | 2.7 | 0.91 | 0.80; 1.04 |
| Kazakhstan (2015) | MICS | 5189 | 7.9 | 2.6 | 1.8 | 1.7 | 1.9 | **0.74** | **0.65; 0.84** |
| Maldives (2016) | DHS | 2511 | 8.6 | 4.8 | 9.9 | 9.6 | 12.4 | **1.19** | **1.07; 1.32** |
| Mexico (2015) | MICS | 7831 | 1.5 | 1.2 | 0.7 | 0.5 | 1.3 | **0.80** | **0.68; 0.94** |
| Montenegro (2018) | MICS | 773 | 5.8 | 0.9 | 1.1 | 1.7 | 1.5 | 0.71 | 0.50; 1.03 |
| Namibia (2013) | DHS | 2608 | 14.3 | 8.8 | 4.2 | 4.0 | 4.5 | **0.72** | **0.63; 0.81** |
| North Macedonia (2011) | MICS | 1297 | 5.5 | 1.9 | 0.3 | 0.1 | 1.6 | **0.64** | **0.45; 0.92** |
| Paraguay (2016) | MICS | 4407 | 2.6 | 0.8 | 0.2 | 1.1 | 0.3 | **0.62** | **0.48; 0.81** |
| Peru (2018) | DHS | 23278 | 0.8 | 0.8 | 0.4 | 0.3 | 0.2 | **0.72** | **0.64; 0.82** |
| Serbia (2014) | MICS | 2363 | 7.3 | 1.9 | 2.2 | 4.8 | 2.9 | 0.87 | 0.72; 1.05 |
| South Africa (2016) | DHS | 1449 | 2.9 | 2.4 | 1.6 | 1.5 | 4.6 | 0.86 | 0.64; 1.14 |
| St Lucia (2012) | MICS | 275 | 9.8 | 2.8 | 0.0 | 2.2 | 4.2 | 0.65 | 0.33; 1.28 |
| Suriname (2018) | MICS | 3378 | 7.6 | 6.7 | 3.7 | 4.7 | 5.1 | 0.90 | 0.81; 1.00 |
| Thailand (2015) | MICS | 11138 | 8.2 | 4.7 | 5.4 | 4.0 | 5.2 | **0.92** | **0.86; 0.98** |
| Turkey (2013) | DHS | 2774 | 5.2 | 1.9 | 0.9 | 0.9 | 0.7 | **0.58** | **0.45; 0.76** |
| Turkmenistan (2015) | MICS | 3708 | 10.4 | 2.5 | 3.4 | 3.3 | 1.4 | **0.63** | **0.54; 0.73** |

**
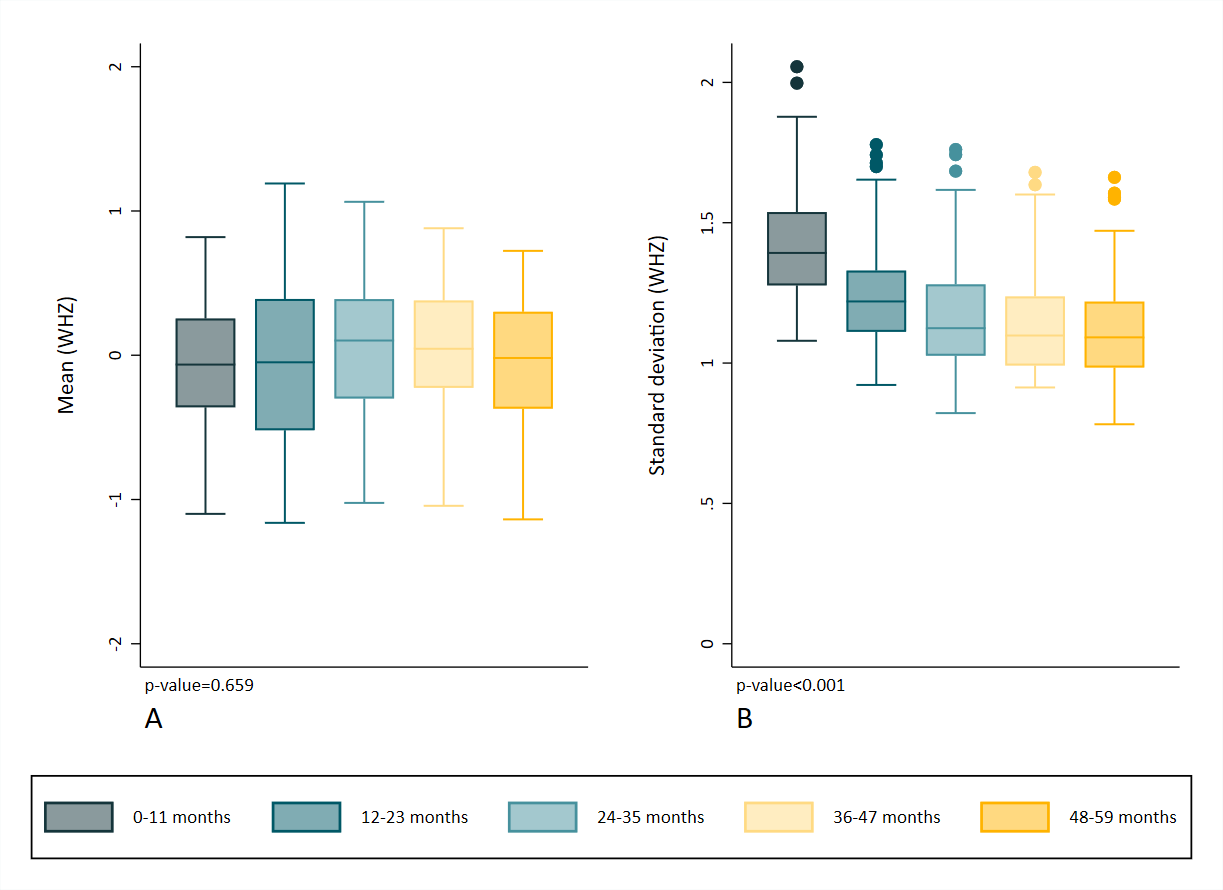
**

**Supplementary Figure 4.** Distributions of mean WHZ (A) and respective standard deviations (B) by age (N=90)

P-value from one-way analysis of variance (ANOVA)

**Supplementary Figure 5.** WHZ mean distribution in the countries with greater decline with age in overweight (A) and wasting (B) prevalence


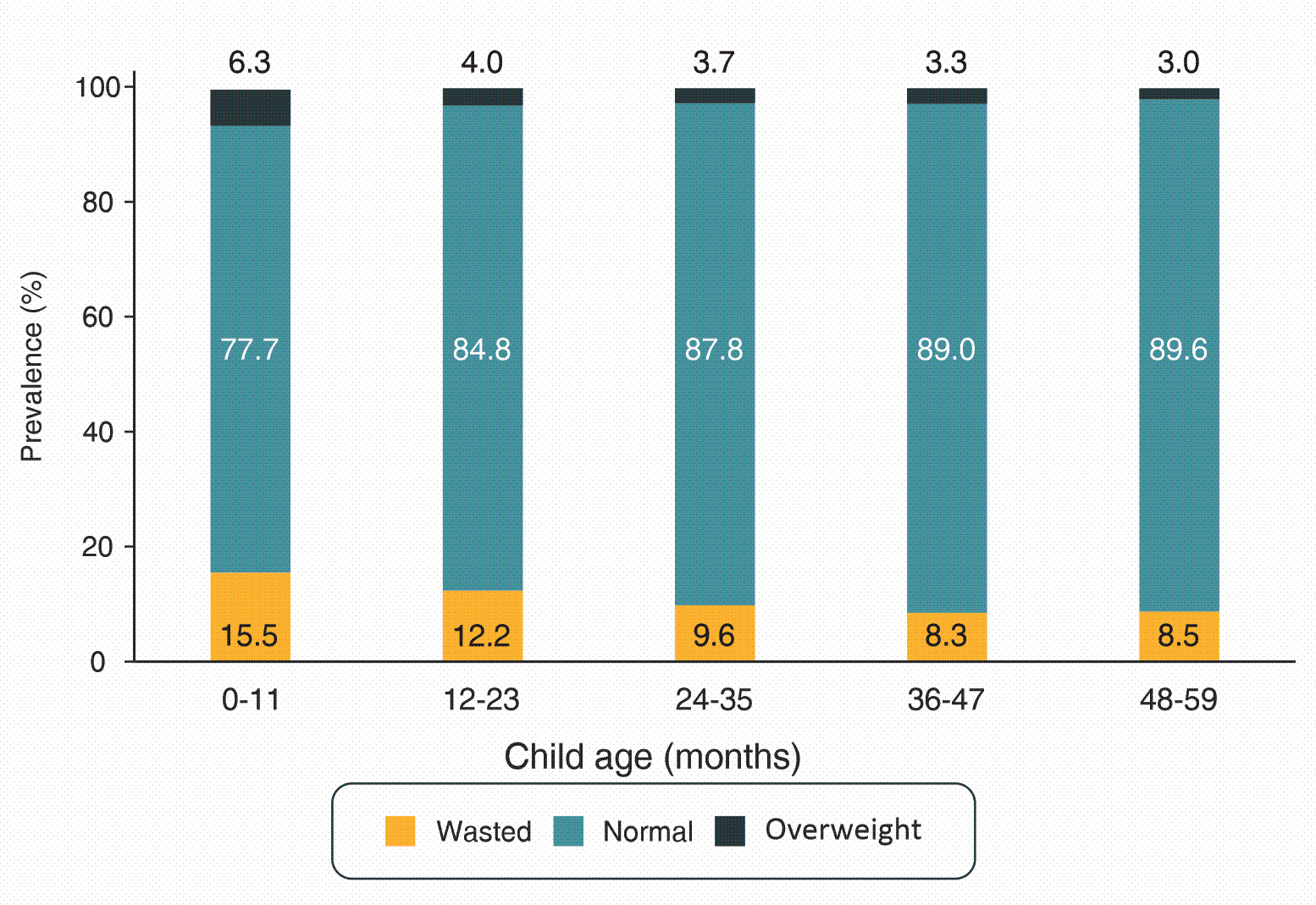


**Supplementary Figure 6.** Prevalence of wasted, normal and overweight children by age in LMIC's (N=90)
